# Effect of different anesthetic agents on left ventricular systolic function assessed by echocardiography in hamsters

**DOI:** 10.1590/1414-431X20165294

**Published:** 2016-08-25

**Authors:** D.M. Tanaka, M.M.D. Romano, E.E.V. Carvalho, L.F.L. Oliveira, H.C.D. Souza, B.C. Maciel, H.C. Salgado, R. Fazan-Júnior, M.V. Simões

**Affiliations:** 1 Departamento de Medicina Interna, Faculdade de Medicina de Ribeirão Preto, Universidade de São Paulo, Ribeirão Preto, SP, Brasil; 2 Departamento de Biomecânica, Medicina e Reabilitação do Aparelho Locomotor, Faculdade de Medicina de Ribeirão Preto, Universidade de São Paulo, Ribeirão Preto, SP, Brasil; 3 Departamento de Fisiologia, Faculdade de Medicina de Ribeirão Preto, Universidade de São Paulo, Ribeirão Preto, SP, Brasil

**Keywords:** Echocardiography, Anesthetics, Systolic function, Hamsters

## Abstract

Determination of left ventricular ejection fraction (LVEF) using *in vivo* imaging is the cardiac functional parameter most frequently employed in preclinical research. However, there is considerable conflict regarding the effects of anesthetic agents on LVEF. This study aimed at assessing the effects of various anesthetic agents on LVEF in hamsters using transthoracic echocardiography. Twelve female hamsters were submitted to echocardiography imaging separated by 1-week intervals under the following conditions: 1) conscious animals, 2) animals anesthetized with isoflurane (inhaled ISO, 3 L/min), 3) animals anesthetized with thiopental (TP, 50 mg/kg, intraperitoneal), and 4) animals anesthetized with 100 mg/kg ketamine plus 10 mg/kg xylazine injected intramuscularly (K/X). LVEF obtained under the effect of anesthetics (ISO=62.2±3.1%, TP=66.2±2.7% and K/X=75.8±1.6%) was significantly lower than that obtained in conscious animals (87.5±1.7%, P<0.0001). The K/X combination elicited significantly higher LVEF values compared to ISO (P<0.001) and TP (P<0.05). K/X was associated with a lower dispersion of individual LVEF values compared to the other anesthetics. Under K/X, the left ventricular end diastolic diameter (LVdD) was increased (0.60±0.01 cm) compared to conscious animals (0.41±0.02 cm), ISO (0.51±0.02 cm), and TP (0.55±0.01 cm), P<0.0001. The heart rate observed with K/X was significantly lower than in the remaining conditions. These results indicate that the K/X combination may be the best anesthetic option for the *in vivo* assessment of cardiac systolic function in hamsters, being associated with a lower LVEF reduction compared to the other agents and showing values closer to those of conscious animals with a lower dispersion of results.

## Introduction

Experimental models of heart disease employing small animals such as rats, mice, rabbits and hamsters have been increasingly used to study the physiopathogenic mechanisms of diseases and to test the effect of new drugs (1). The hamster, in particular, attains special interest as a model of chronic dilated cardiomyopathy, including Chagas' disease (2,3).

In parallel to the expansion of areas of interest in preclinical research, advances of *in vivo* cardiac imaging ([Bibr B04] [Bibr B05] [Bibr B06] [Bibr B07] [Bibr B08]–9) have permitted noninvasive assessment of cardiac structure and function in a reproducible and satisfactory manner. In view of its practicality, low cost and wide availability, echocardiography is the imaging method most frequently used (10), permitting the sequential assessment of morphological remodeling parameters and of systolic and diastolic left ventricular function (11). Among the parameters that can be measured by echocardiography, the left ventricle ejection fraction (LVEF) is the most frequently used to determine left ventricle (LV) systolic function both in the preclinical (12) and clinical scenarios (13).

Although echocardiography can be applied to both anesthetized and conscious animals (14,15), the use of anesthetic agents permits a more reliable and reproducible image collection without causing stress or pain to the animal.

Several anesthetic agents have been used to sedate small rodents during echocardiography, such as the combination of ketamine and xylazine (5,16), pentobarbital sodium (17), thiopental (18), isoflurane (6), and halothane (19). However, several studies have demonstrated that these substances may interfere with cardiac function (4,19), with relevant controversy about the magnitude of the changes in LVEF induced by different anesthetics in various animal species (5,6,20).

Thus, the objective of the present study was to assess the effects of different anesthetics on LV systolic function in healthy hamsters using transthoracic echocardiography.

## Material and Methods

### Animals and experimental protocol

Fifteen 12-week-old female hamsters (*Mesocricetus auratus*) (Anilab; Animais de Laboratório Criação e Comércio Ltda., Brazil) were used for the study. The animals were kept in climatically controlled housing on a 12-h light/dark cycle in the animal facilities of the Faculdade de Medicina de Ribeirão Preto, with free access to water and a standard diet.

### General study design

Conscious animals were submitted to sequential acquisition of echocardiographic images under the effect of different anesthetic agents. The images were acquired at 1-week intervals in the following sequence: 1) unanesthetized conscious animals; 2) animals anesthetized with isoflurane (ISO); 3) animals anesthetized with thiopental (TP), and 4) animals anesthetized with ketamine and xylazine (K/X). A 1-week interval between studies was chosen in order to guarantee a long washout time of the effect of the anesthetics used, so that the effect of an anesthetic employed in one session would not contaminate the results of the subsequent session.

### Anesthetic procedure

For the imaging of conscious animals, the hamsters were trained for 2 consecutive days before the examination. Training consisted of holding the animal for 5 min in the position required for the exam, applying the ultrasound gel and touching the animal's chest with the probe to simulate the exam.

Isothane (Baxter Healthcare of Puerto Rico, Porto Rico) was used at 5% concentration for 1 min for the induction of anesthesia and was then inhaled through a nasal cone with an oxygen flow of 3 L/min at 1.5% concentration for the maintenance of anesthesia.

Thiopental (Cristália, Produtos Químicos Farmacêuticos Ltda., Brazil) was injected intraperitoneally as a single dose of 50 mg/kg.

The combination of ketamine hydrochloride (Ketamina Agener, União Química Farmacêutica Nacional S/A, Brazil) and xylazine (Dopaser, Laboratórios Calier S/A, Spain) was injected intramuscularly as a single dose of 100 and 10 mg/kg, respectively.

The occurrence of the expected depth of anesthesia was determined when the animals gave a minimal motor response to pinching of a paw.

### Echocardiogram

After sedation, the anterior region of the animals’ chests was depilated by applying a commercial human depilatory cream. The animals were positioned on left lateral decubitus under spontaneous ventilation and the echocardiogram was obtained using the Philips HD11XE (Best, DA, Netherlands) high-resolution two-dimensional echocardiography system equipped with a linear trans-ducer at the frequency of 15 MHz. The parasternal window was used to obtain the long and short LV axes at the level of the papillary muscles. Images were recorded in M mode to measure the thickness of the interventricular septum and of the posterior LV wall, as well as their systolic and diastolic dimensions.

The images obtained were recorded for later off-line analysis. An echocardiographer experienced in obtaining and analyzing images of small animals and blind to the animal groups analyzed the images. The dimensions of the LV obtained during diastole (LVdD) and systole (LVsD) were used to calculate the ejection fraction using ventricular volumes estimated by the Teichholz method, according to the following formula: (LVEF) = (LVdV) – (LVsV) systolic V/(LVdV) diastolic V × 100. Each volume was calculated from Teichholz method with linear dimension (D) of the LV as follows: V = (7/2.4 D) × D^3^.

The lower normal limit of LVEF was calculated by subtracting two standard deviations from the mean obtained for conscious animals.

Left ventricle fractional shortening (LVFS) was calculated from diastolic and systolic ventricle diameters. The measurements are reported as the mean of five consecutive cardiac cycles using the same projection, transducer position and angulation in the same frozen image frame. The heart rate (HR) of the animals was also recorded during the echocardiography exam.

### Ethical aspects

The project was approved by the System of the National Council for the Control of Animal Experimentation of our institution (protocol No. 064/2014).

### Statistical analysis

Continuous variable data are reported as means±SE and nominal variables are reported as absolute (n) and relative (%) frequency. The Kolmogorov-Smirnov test was used to determine whether the sample distribution of a given variable was Gaussian. Parametric paired tests for multiple measures (repeated measures ANOVA) were used for the simultaneous comparison of the variables means. A 5% two-tailed level of significance (P<0.05) was considered for all analyses.

The coefficient of variation (CV% = standard deviation/mean) was calculated to determine the dispersion of the results, and the F test was used to compare the variance between 2 variables.

## Results

Three of the 15 animals in the initial sample were excluded because they exhibited images of suboptimal quality for analysis. The mean weight of the 12 remaining animals was 148.7±8.2 g. Table 1 summarizes the echocardiography results.

**Table:**
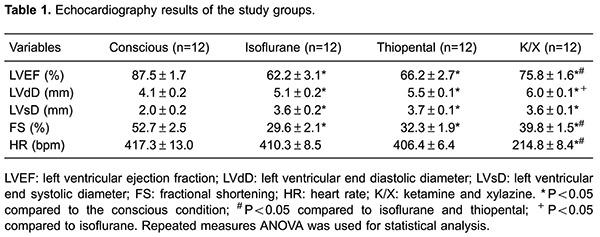

**Figure 1:**
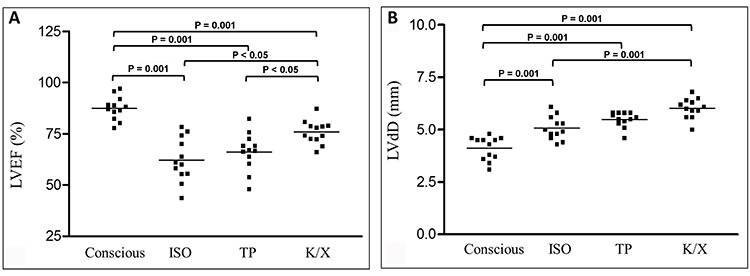
*A*, Left ventricular ejection fraction (LVEF) assessments of conscious and anesthetized animals; *B*, left ventricular end diastolic diameter (LVdD) of conscious and anesthetized animals. ISO: isoflurane; TP: thiopental; K/X: ketamine/xylazine. Repeated measures ANOVA was used for statistical analysis.

The LVEF obtained for anesthetized animals was significantly lower than the values obtained for the conscious animals P<0.001. In addition, the use of ISO and TP was associated with significantly lower LVEF values than when using K/X (P<0.001 and P<0.05, respectively; Figure 1A).

**Figure 2:**
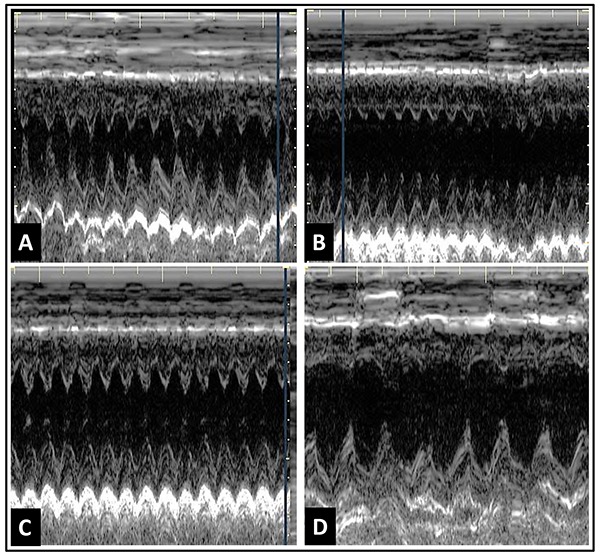
Representative echocardiogram M-mode images of *A*, conscious animals (left ventricular ejection fraction (LVEF)=91.9%) and animals anesthetized with *B*, isoflurane (LVEF=61%); *C*, thiopental (LVEF=82.4%); and *D*, ketamine/xylazine (LVEF=87.2%).

The Figure 2 shows the ventricular function by M mode of an animal when conscious and under the effect of the tested anesthetic agents.

When anesthetized with K/X, the animals showed more homogeneous results with less dispersion of LVEF values around the mean and with lower CV (7.52%) than animals anesthetized with ISO (17.2%, F test=3.517, P=0.02) and TP (13.9%, F test=2.607, P=0.06).

Individual LVEF values with the use of ISO ranged from 43.5 to 78.3%, with 83% of the animals exhibiting LVEF values below the lower normal limit obtained with conscious animals (lower limit value = 76.0%). With TP, 92% of the animals showed LVEF values below the lower normal limit. With the use of K/X, only 50% had values below the normal limit, in addition to presenting a lower CV than the other groups.

Similarly, the shortening fraction values were also higher in conscious animals (52.7±2.5%) than in those anesthetized with ISO (29.6±2.1%), TP (32.3±1.9%) and K/X (39.8±1.5%), P<0.0001. Among the anesthetics, K/X elicited higher shortening fraction values than ISO (P<0.01) or TP (P<0.05).

Analysis of the systolic LV dimensions revealed that conscious animals had significantly lower values in the LVsD (2.0±0.2 mm) than animals anesthetized with ISO (3.6±0.2 mm), TP (3.7±0.1 mm) and K/X (3.6±0.91 mm), P<0.0001.

Regarding the LVdD, conscious animals also had lower values (4.1±0.2 mm) than animals anesthetized with ISO (5.1±0.2 mm), TP (5.5±0.1 mm) and K/X (6.0±0.1 mm), P<0.001. Comparison of the different anesthetics revealed that K/X elicited higher LVdD values than ISO (P<0.001), Figure 1B.

**Figure 3:**
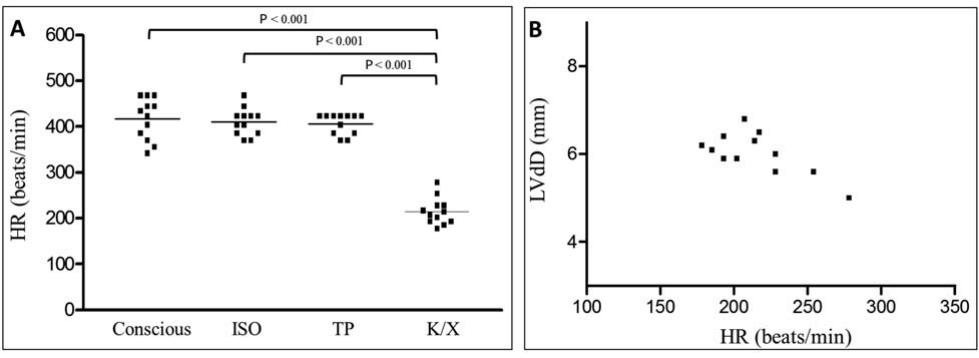
*A*, Individual values for heart rate (HR) of conscious and anesthetized animals. ISO: isoflurane; TP: thiopental; K/X: ketamine/xylazine. Repeated measures ANOVA. *B*, Dispersion plot showing the correlation between HR and left ventricular end diastolic diameter (LVdD) of animals anesthetized with K/X. Linear (Pearson) correlation was used for statistical analysis.

The HR of conscious animals was 417.3±13.0 bpm, a value comparable to that obtained for animals anesthetized with ISO (410.3±8.5 bpm) and TP (406.4±6.4 bpm), P>0.05. HR values were significantly lower for the animals anesthetized with K/X (214.8±8.4 bpm), P<0.001, than for those in the other three conditions (Figure 3A).

A significant negative correlation was also observed between HR and LVdD when the animals were anesthetized with K/X (r =-0.7; P=0.01), Figure 3B.

Intra-observer variability of LVEF echocardiography measurements was adequate, with a mean difference between 2 measurements of 1.5 ejection units (confidence interval of -8.6 to 11.7). All paired measures were within limits according to a Bland-Altman analysis.

## Discussion

In the present study, we used echocardiography to assess LV function in a group of animals assessed in the conscious state and also submitted to commonly employed anesthetic agents. The main results showed that the use of any of the tested anesthetic agents induced a reduction of LVEF compared to the same animals in the conscious state.

In addition, we observed a significant heterogeneity of the effects between the anesthetics on left ventricle systolic function (LVSF), with the K/X combination showing the lowest LVEF reduction. Even more important, anesthesia with K/X was associated with a lower dispersion of individual LVEF values, which was similar to that observed when the animals were assessed in the conscious state. A greater variation of the individual measurements was observed in the animals anesthetized with the other drugs.

Previously published studies assessing the effects of anesthetic agents on cardiac function in small rodents have shown conflicting results, especially when the images are acquired *in vivo.*

Considering that LVEF may represent the primary outcome in experimental models of heart disease, information about the effects of different anesthetics on the investigated animal species is of primordial importance. In this regard, useful models of stable chronic heart failure have been described in hamsters (2,3). For the present study, an extensive search of the specialized literature did not detect reports assessing the effects of different anesthetics on LVSF in hamsters using imaging methods *in vivo.*

The present results show that all the anesthetics used elicited a lower LVEF compared to that obtained for conscious animals. It is relevant to consider that these results could be attributed not only to a depressant effect of the anesthetic agents over the cardiac systolic function, but also to the relative LVEF increase in conscious animals during the echocardiogram. An increased sympathetic stimulus secondary to the stress imposed on the animals during image acquisition may lead to a hyperdynamic cardiac state and to an LVEF increase above the values expected for a true situation of rest. Indeed, previous studies have demonstrated sympathetic hyperactivity in conscious animals submitted to an echocardiogram (14,21). However, this mechanism remains a hypothesis, as we did not collect data to confirm it. It should be pointed out that, as recommended by current guidelines for reducing the stress imposed to animals (14), the results were obtained after the animals had been trained in 2 simulated echocardiography sessions before the final image acquisition.

In the present study, we used LVEF by the Teichholz method, and LVFS to evaluate the effects of anesthetics in echocardiogram results. It is relevant to mention that the use of other methods, like fractional area changing or ejection fraction with direct volume determination by the Simpson method, to measure left ventricle systolic function would be complementary, but these methods are not appropriate for small animals. On the other hand, since the animals presented no left ventricle abnormalities, additional measurements would probably yield similar results regarding the effects of the different anesthetic agents.

Our results also showed that the use of K/X was associated with the smallest LVEF reduction compared to the other agents, eliciting values closer to those obtained for conscious animals. This effect involved a greater reduction of HR and a marked increase of LVdD. Although studies on the effects of anesthetics on LV systolic function compared to the K/X combination have been conducted predominantly on mice, the results described in the present investigation are in agreement with several reports (5,14).

Hart et al. (5) detected a reduction of HR in mice anesthetized with K/X compared to those anesthetized with tribromoethanol. However, LVdD and LVFS were greater in the K/X group, suggesting that, despite having a negative chronotropic effect, K/X had no adverse effects on the echocardiography indices of LV ejection function. In addition, the authors considered that the better systolic function obtained with K/X might have been associated with the increased preload (LVdD) and recruitment of the contractile force of the LV reserve. This was also valid for our results, which showed a significant negative correlation between individual HR and LVdD values in the animals anesthetized with K/X. Thus LVdD enlargement occurs as a volumetric adaptation to a more prolonged diastole and does not represent a depressed systolic function, which was preserved when LVEF was assessed. In addition, the significant HR reduction observed in the animals anesthetized with K/X agrees with previous studies (15,19).

One of the few studies investigating the effect of anesthetics on hamsters used *in vitro* methods to assess the effects of ketamine on the intrinsic contractility of the papillary muscles of healthy hamsters and of hamsters with heart disease. The results showed that ketamine had positive inotropic effects on healthy animals, mainly owing to increased calcium influx into the cardiomyocytes, but less pronounced effects on animals with cardiomyopathy (22). In this same experimental setting, the effects of halothane and isoflurane were tested (23). Both anesthetics had negative inotropic effects that were more marked in the animals with cardiomyopathy than in healthy animals. These anesthetics also induced changes in left ventricle relaxation when injected in high doses. Other reports also indicate that halogenated anesthetic agents have a depressive effect on the myocardium, observed both *in vivo* and *in vitro* (24,25), due to changes in intracellular calcium homeostasis and to the reduced function of the sarcoplasmic reticulum (26). Thus, our results, which showed higher LVEF values with the use of K/X than with the other anesthetics such as ISO and TP, are in agreement with these *in vitro* studies conducted on animal models.

Another relevant aspect observed in the present study was the variation of the individual measurements of LVEF. The dispersion of LVEF values was significantly higher with the use of ISO and TP than with the use of K/X. It should be pointed out that this value dispersion has not been considered often in previous reports, and it may represent an important source of variation in the results of different experimental models.

Our results indicated that the K/X combination seems to be the best anesthetic option for the *in vivo* assessment of the LV systolic function in hamsters since it was associated with a lower reduction of LVEF compared to the other agents. LVEF values with this agent were similar to those observed in conscious animals and had a lower individual dispersion.

### Limitations

A limitation of the present study was the lack of arterial blood pressure (BP) assessment that could have been useful to correlate the LVEF values to the afterload changes in the several experimental conditions. However, a literature review on hemodynamic studies demonstrated that virtually all anesthetic agents lead to a reduction in BP compared to the conscious condition, and ISO is associated to lower BP reductions than other agents (27,28). Also, invasive measures of cardiac output were not applied and should be integrated with echocardiography measures in future tests of anesthetic cardiovascular effects in rodents.
